# Analysis of prostatic spindle cell lesions: Three case reports and literature review

**DOI:** 10.1097/MD.0000000000043768

**Published:** 2025-08-15

**Authors:** Quanxi Wang, Peng Li, Zhengdong Zong, Ke Dou

**Affiliations:** a School of Medicine, University of Electronic Science and Technology of China, Chengdu, China; b Department of Urology, Sichuan Provincial People’s Hospital, University of Electronic Science and Technology of China, Chengdu, China.

**Keywords:** diagnosis, sarcoma, spindle cell lesions of the prostate, treatment

## Abstract

**Rationale:**

Spindle cell lesions of the prostate originate from the specialized stroma or prostate epithelium. At present, these lesions are relatively rare, but the histomorphological features they involve are extremely diverse, which can easily lead to misdiagnosis in the clinical diagnosis process. This study reports on 3 cases of different types of spindle cell lesions of the prostate, with the aim of providing valuable reference information for clinicians when dealing with such cases, to help them make more accurate and effective clinical decisions.

**Patient concerns:**

A retrospective analysis of the clinical data of 3 patients diagnosed with different spindle cell lesions of the prostate at the Sichuan Provincial People’s Hospital, Sichuan Academy of Medical Sciences, from January 2023 to October 2024. All 3 patients underwent transperineal prostate biopsy with 12 needles. Two patients were diagnosed with the assistance of fluorescence in situ hybridization (FISH).

**Diagnoses:**

Based on pathological examination and immunohistochemical testing results, 1 patient was diagnosed with nodular hyperplasia of prostatic interstitial smooth muscle. The other 2 patients who underwent FISH testing were diagnosed with prostatic stromal sarcoma in one case and prostatic synovial sarcoma in the other.

**Interventions:**

One patient elected to undergo transurethral holmium laser-induced prostatectomy. Another patient received targeted therapy with anlotinib. The third patient received liposome doxorubicin combined with ifosfamide chemotherapy.

**Outcomes:**

For the cases of nodular hyperplasia of prostatic interstitial smooth muscle, no signs of recurrence have been found so far. The patient with prostatic stromal sarcoma unfortunately passed away after 3 months of follow-up. However, during the subsequent observation period, the tumor volume of patients with synovial sarcoma of the prostate exhibited a reduction, and no further deterioration was noted.

**Lessons:**

Spindle cell lesions of the prostate have diverse histological manifestations. A diagnosis can only be confirmed through the results of a biopsy and immunohistochemistry. Classification of some tumors should also be diagnosed with the assistance of FISH. Once diagnosed, timely intervention is required, including surgery, adjuvant radiotherapy, adjuvant chemotherapy, targeted therapy, etc. Malignant tumors, in particular, progress rapidly and have a poor prognosis, so a detailed treatment plan needs to be formulated.

## 1. Introduction

Spindle cell lesions of the prostate are a relatively rare type of disease. Lesions can be broadly classified into 2 categories, benign and malignant. According to their origin, they are divided into specialized prostatic stromal lesions and prostatic epithelial lesions. The former includes hyperplastic nodules of the prostatic stroma, stromal tumors of uncertain malignant potential (STUMP), and prostatic stromal sarcoma. The latter includes leiomyoma, solitary fibrous tumor, gastrointestinal stromal tumor, inflammatory myofibroblastic tumor, prostatic synovial sarcoma, rhabdomyosarcoma, etc.^[1]^ Due to its complex classification, a definitive diagnosis cannot be made based on the clinical manifestations, laboratory tests, or imaging tests. The final diagnosis must rely on the results of pathological examination combined with immunohistochemical testing, and may even require fluorescence in situ hybridization (FISH) genetic testing. This study retrospectively analyzed the clinical data of 3 patients diagnosed with different types of spindle cell lesions of the prostate at Sichuan Provincial People’s Hospital from January 2023 to October 2024. It provides a detailed description of the clinical manifestations, diagnostic strategies, treatment options and prognosis of different types of spindle cell lesions of the prostate, with the aim of providing a valuable reference for clinicians when dealing with such cases and promoting the formulation of more accurate and effective clinical decisions.

## 2. Materials and methods

**Figure 1.**
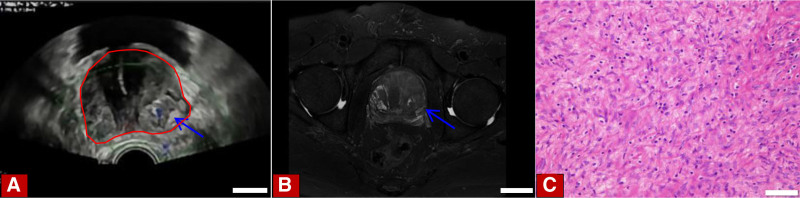
(A) The transrectal ultrasound of the prostate shows that the prostate has a triangular shape with an enlarged volume (the part circled in red), and a high-echogenic nodule is detected within the left parenchyma (blue arrow), the scale in the figure is 2 cm. (B) MRI suggests an enlarged prostate with high signal in the T2WI fat-suppression sequence (blue arrow), the scale in the figure is 3 cm. (C) Histological features are mild proliferation of short spindle-shaped cells (HE staining, ×40). MRI = magnetic resonance imaging, HE = hematoxylin and eosin, T2WI = T2-weighted images.

**Figure 2.**
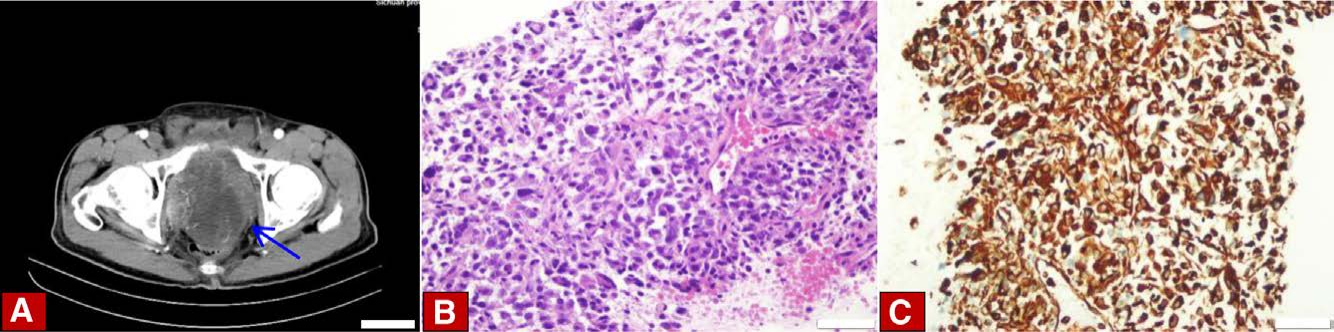
(A) CT revealed a mass in the prostate with an irregular shape and uneven enhancement on the enhanced scan, with unclear boundaries with the bladder and surrounding soft tissues (blue arrow), the scale in the figure is 5 cm. (B) The histological features show spindle cell proliferation with significant cellular atypia (HE staining, ×40). (C) The Vimentin expression was positive (immunohistochemical staining, ×40). CT = computed tomography, HE = hematoxylin and eosin.

**Figure 3.**
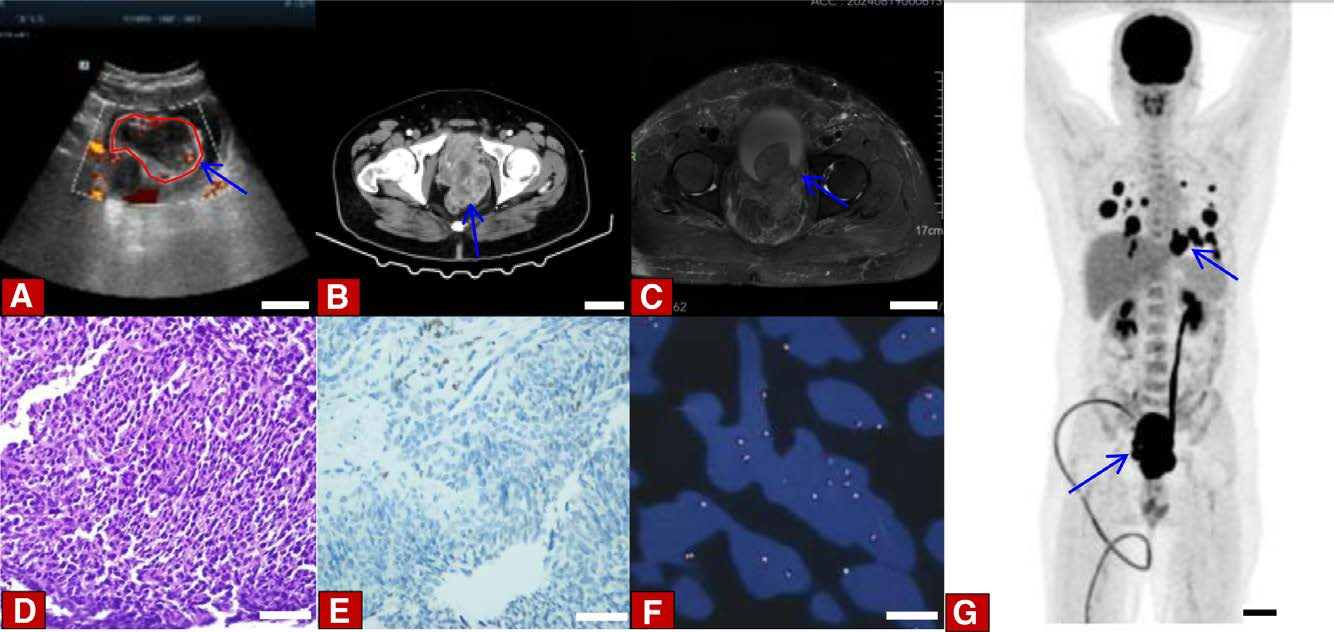
(A) Transrectal ultrasound examination shows abnormal prostate morphology, enlarged volume (blue arrow), and uneven echogenic mass extending into the bladder (the part circled in red), the scale in the figure is 3 cm. (B) CT reveals an enlarged prostate with indistinct boundaries with the rectum, bladder, seminal vesicles, left anal sphincter, and adjacent pelvic wall. The enhancement scan has obvious uneven enhancement (blue arrow), the scale in the figure is 3 cm. (C) MRI suggests a space-occupying lesion on the posterior inferior wall of the bladder and within the prostate. T2WI fat-suppression sequence shows high signal (blue arrow), the scale in the figure is 5 cm. (D) The pathological features are characterized by abundant tumor cells that are spindle-shaped and arranged in interwoven fascicles (HE staining, ×40). (E) Tumor cells were positive for TLE-1 expression (immunohistochemical staining,×40). (F) The FISH results detected the separation of red and green signals and a break in the SS18 gene (×40). (G) PET-CT revealed an irregular soft tissue density mass in the prostate area and significantly active FDG metabolism in multiple parts of the body (blue arrow), the scale in the figure is 5 cm. CT = computed tomography, FDG = fluorodeoxyglucose, FISH = fluorescence in situ hybridisation, HE = hematoxylin and eosin, MRI = magnetic resonance imaging, PET-CT = positron emission tomography-computed tomography, TLE-1 = transducer-like enhancer of split 1, T2WI = T2-weighted images.

We retrospectively analyzed the clinical data of 3 patients diagnosed with different spindle cell lesions of the prostate at Sichuan Provincial People’s Hospital from January 2023 to October 2024. The first patient was a 69-year-old elderly man who was admitted to the hospital with lower urinary tract symptoms dominated by dysuresia. Other symptoms included tachyuria, frequent micturition, acraturesis, and a feeling of incomplete bladder emptying. Digital rectal examination of the patient shows an enlarged prostate, the central furrow becomes shallower, and no nodules were touched. The blood test results showed a total prostate-specific antigen (PSA) of 11.4 ng/mL. Transrectal ultrasound of the prostate shows that the prostate has a triangular shape and is about 5.7 × 5.2 × 5.8 cm^3^ in size (Fig. 1A). Magnetic resonance imaging (MRI) suggests an enlarged prostate. T1-weighted images (T1WI) show low signal, T2-weighted images (T2WI) show high signal, diffusion-weighted imaging (DWI) demonstrates an equal or slightly high signal, enhanced scanning reveals persistent nodular enhancement, while the fat-suppression sequence exhibits a high signal (Fig. 1B). We performed a prostate needle biopsy on the patient and obtained a total of 12 tissue samples, of which 11 were benign prostate tissue and 1 tissue was a focus of small glands with minimal atypia suspicious for malignancy (Fig. 1C). Based on the preoperative examination, the patient was considered to have benign prostatic hyperplasia, and surgical contraindications were ruled out. The patient underwent transurethral holmium laser-induced prostatectomy under general anesthesia. The patient recovered well after the operation and was discharged from the hospital smoothly. The second patient is a 70-year-old male elder, who was hospitalized mainly due to dysuresia, accompanied by symptoms of tachyuria and frequent micturition. The digital rectal examination indicated an enlarged prostate volume, with no nodules being touched. The results of the blood test revealed that the total PSA level was 1.09 ng/mL. The abdominal computed tomography (CT) reveals an irregularly shaped mass within the prostate, exhibiting uneven enhancement upon contrast administration, and demonstrating indistinct margins with the bladder and adjacent soft tissues (Fig. 2A). The MRI from the patient’s previous hospitalization reveal a deviation from the normal prostate structure, featuring an irregularly shaped soft tissue mass that emits mixed signals, it is about 10.5 × 9.5 × 10 cm^3^ in size. It exhibits low signal intensity on T1WI, shows high signal intensity on T2WI, and on fat suppression sequences, it presents with high signal intensity. The DWI shows mixed high signal intensity. There is significant heterogeneous enhancement on contrast-enhanced scanning. The bilateral seminal vesicles are not clearly visible, and the bladder and rectum are compressed.We performed a prostate biopsy on the patient, which showed spindle cell hyperplasia with atypia. The immunohistochemical diagnostic results indicate a mesenchymal origin malignant tumor (Fig. 2B, C). No amplification of CDK4/MDM2 genes and no classical meaningful SS18 gene translocation were detected in FISH. The diagnosis is comprehensively considered to be prostatic stromal sarcoma. The patient did not undergo a whole-body positron emission tomography-computed tomography (PET-CT) scan due to personal reasons. After a comprehensive discussion, it was considered that the patient’s tumor was very large, highly malignant, infiltrating surrounding tissues, and the surgery would be difficult. It was suggested to start with radiochemotherapy. The oncology department recommended liposome doxorubicin chemotherapy combined with anlotinib targeted therapy. However, the patient declined chemotherapy and opted for anlotinib targeted therapy instead. The final patient was a 36-year-old young male who was hospitalized for treatment primarily due to dysuresia, accompanied by symptoms such as acraturesis, hesitancy to urinate, and increased frequency of urination at night. Digital rectal examination revealed a coarse surface of the prostate, significant enlargement, a pliable texture, and palpable suspicious nodules. The blood examination results indicated a total PSA of 6.33 ng/mL. Transrectal prostate ultrasound revealed an abnormal prostate shape, measuring approximately 7.2 × 10.3 × 7.8 cm^3^, and detected an irregular echogenic mass conducting into the bladder (Fig. 3A). The abdominal CT shows an enlarged prostate volume with abnormal shape, unclear boundaries with surrounding tissues, and significantly uneven enhancement on contrast scan (Fig. 3B). The MRI revealed mass-like lesions on the posterior inferior wall of the bladder and within the prostate. These lesions presented with high signal intensity on T1WI, whereas the signal intensity was heterogeneous on T2WI, and on DWI, there were signs of restricted diffusion. The left posterior margin of the prostate invaded the rectal mesentery, and the adjacent left pelvic floor muscles were compressed. Additionally, a high signal nodule protruding outward was visible on the right anterior wall of the bladder on T2WI, and the fat suppression sequence also showed high signal (Fig. 3C). We performed a prostate biopsy on the patient, and the results indicated the presence of a spindle cell tumor. The immunohistochemical analysis was consistent with spindle cell sarcoma, but the possibility of synovial sarcoma cannot be completely ruled out yet (Fig. 3D, E). Therefore, we conducted FISH testing on the patient, and the results showed separated red and green signals, with the gene break-apart rearrangement test being positive (Fig. 3F). After comprehensive examination, we diagnosed him with synovial sarcoma of the prostate. The patient does not exclude the possibility that a distant metastasis has occurred, this was also confirmed on PET-CT. The patient showed an irregular soft tissue density mass shadow in the prostate area with unclear boundaries on PET-CT. A soft tissue density nodule was seen in the right front of the bladder, and multiple solid nodules of various sizes were seen in both lungs, with significantly active fluorodeoxyglucose metabolism (Fig. 3G). The patient has now developed multiple metastases throughout the body. Surgical treatment is not currently considered, and a comprehensive antitumor treatment strategy is recommended. After comprehensive discussion, it was decided that the chemotherapy regimen for the patient would be a combination of Liposome doxorubicin and ifosfamide. Clinical data on 3 patients with prostatic spindle cell lesions (Table 1).

**Table 1:** Clinical data with prostatic spindle cell lesions.

Cases	Age (yr)	Symptom	PSA (ng/mL)	PC	SP	CT	TT	Prognosis
1	69	Dysuresia	11.4	NHPISM	TULIP	UF	UF	NR
2	70	Dysuresia	1.09	PS	UF	UF	ANL	DOD
3	36	Dysuresia	6.33	PSS	UF	LD+IFO	UF	NP

ANL = anlotinib, CT = chemotherapy, DOD = dead of disease, LD+IFO = liposome doxorubicin+ifosfamide, NHPISM = nodular hyperplasia of prostatic interstitial smooth muscle, NP = no progress, NR = no recurrence, PC = pathological classification, PS = prostatic stromal sarcoma, PSA = prostatic specific antigen, PSS = prostatic synovial sarcoma, SP = surgical procedure, TT = targeted therapy, TULIP = transurethral holmium laser-induced prostatectomy, UF = unfinished.

## 3. Results

The pathological specimens of the 3 patients were processed according to standardized histopathological procedures. First, they were fixed with 10% formalin, followed by efficient dehydration using a closed automated tissue dehydrator. After paraffin embedding, sections were prepared and stained with hematoxylin and eosin for routine histological examination. Subsequently, immunohistochemical staining was performed. In the surgical specimen of the first patient, pathological examination revealed hyperplasia of spindle cells in the prostate. The examination results showed not only hyperplasia of spindle cells but also atypical features in the pathological specimen of the second patient. As for the last patient, the prostate biopsy results indicated the presence of a spindle cell tumor. Immunohistochemical staining for CK and CD34 was positive in the specimen of the first patient, while no positive reactions were observed in the other 2 patients. SMA staining was positive in all 3 patients, and the Ki67 proliferation indices were recorded as 2%, 30%, and 60%, respectively. Regarding FISH testing, no abnormalities were found in the second patient, while the results for the third patient suggested the presence of gene breakage and recombination. Based on detailed examination results, 3 patients were diagnosed with nodular hyperplasia of prostatic interstitial smooth muscle, prostatic stromal sarcoma, and prostatic synovial sarcoma, respectively. After a period of continuous follow-up observation, the first patient currently shows no signs of recurrence. In contrast, the second patient unfortunately passed away just 3 months after the follow-up. As for the last patient, disease stabilization under chemotherapy over 2 months, pending surgical reassessment. Immunohistochemical results of 3 patients with prostatic spindle cell lesions (Table 2).

**Table 2:** Immunohistochemical results of prostatic spindle cell lesions.

PC	AR	CK	CD34	SMA	PR	S-100	EMA	TLE1	INI1	Myogenin	Desmin	Vimentin	Ki67 (%)	Key markers
NHPISM	+	+	+	+	−	−	NA	NA	NA	NA	+	−	2	CK, CD34,SMA
PS	−	−	−	+	+	−	−	+	+	−	+	+	30	CD34, PR, vimentin
PSS	+	−	−	+	+	−	+	+	+	−	−	NA	60	CK, EMA, TLE1

AR = androgen receptor, CD = cluster of differentiation, CK = cytokeratin, EMA = epithelia membrane antigen, INI1 = integrase interactor 1, NA = not abailable, NHPISM = nodular hyperplasia of prostatic interstitial smooth muscle, PC = pathological classification, PR = progesterone receptor, PS = prostatic stromal sarcoma, PSS = prostatic synovial sarcoma, SMA = smooth muscle actin, TLE1 = transducer-like enhancer of split 1.

## 4. Discussion

Spindle cell lesions of the prostate have a variety of clinical manifestations, and there are usually no specific symptoms. Some patients may accidentally discover this disease when they seek medical treatment for other prostate diseases. The 3 patients we observed all had symptoms related to urination, mainly including frequent micturition, tachyuresis, urinary hesitancy, acraturesis, and increased nighttime urination. These symptoms are basically the same as those of benign prostatic hyperplasia, which can easily lead to misdiagnosis of the disease and delay treatment. Digital rectal examination is the most important physical examination, which can assess the volume, texture, hardness, and surface condition of the prostate. All 3 patients underwent this examination, and the results showed that the prostate volume was increased in 2 patients, accompanied by a shallowing of the central sulcus. The third patient exhibited a rough and enlarged prostate surface with suspicious nodules, which may indicate malignant lesions. In terms of serological tests, the PSA levels of the 3 patients were 11.4 ng/mL, 1.09 ng/mL, and 6.33 ng/mL, respectively (normal PSA value <4 ng/mL). According to the results, we can notice that the PSA levels of the first and third patients are elevated, while the PSA level of the second patient is within the normal range. PSA is synthesized and secreted by epithelial cells of the prostate. It is more sensitive to epithelial-derived prostate cancer, but may be within the normal range for tumors of stromal origin.^[2]^ No biochemical indicators with high sensitivity and specificity have been found in laboratory tests for screening for spindle cell lesions of the prostate.^[3]^

Nonspecific symptoms often require further diagnostic steps, such as imaging examinations. Transrectal ultrasound is able to help assess the size of the prostate, clearly revealing the internal structure of the prostate and its relationship with the surrounding organs.Combined contrast-enhanced ultrasound technology can further improve the accuracy of diagnosis of prostate diseases.^[4]^ In our first and third patients, ultrasound revealed an increase in prostate volume, and the third patient in particular also showed an abnormal prostate shape. In the CT evaluation of the second and third patients, the prostate was enlarged and irregular in shape, with blurred boundaries with the surrounding soft tissues, which suggested the possibility of a tumorous lesion. The whole-body PET-CT scan of the third patient showed an irregular soft tissue density mass in the prostate area, and fluorodeoxyglucose metabolism was significantly active in multiple parts of the body, indicating that distant metastases had occurred. Compared to ultrasound, CT examination is more valuable in determining the location, nature, degree of infiltration of adjacent organs, and staging of the tumor. Moreover, PET-CT has significant indicative value for distant metastasis of tumors.^[5,6]^ The MRI of the 3 patients all revealed an enlarged prostate with high signal on T2WI. DWI and fat-suppression sequences displayed high signal in all cases, and all 3 patients showed signs of compression of surrounding tissues. The third patient had lymph node involvement in the left iliac paravertebral and bilateral inguinal regions, suggesting that the lesion had already spread to the lymph nodes. Studies have shown that in the diagnosis of prostatic sarcoma, MRI may appear as an even low, equal or high signal on T1WI, and an uneven equal, slightly high or high mixed signal on T2WI. In enhanced scanning, the lesion area may appear as a mass of unevenly enhanced areas, while DWI shows restricted diffusion and a decreased apparent diffusion coefficient value.^[7]^ These findings are consistent with our observations. Compared with CT, MRI can more clearly depict the relationship between the tumor and surrounding tissues and the invasion of adjacent structures from multiple angles and levels. Therefore, it is considered as the preferred method of imaging examination for this disease.^[8,9]^

None of the above examinations can make a definitive diagnosis of the lesion. A final diagnosis must be based on the results of pathological examination, immunohistochemical staining, or even genetic testing. The histological features of stromal nodules of hyperplasia of the prostate are multiple nodular hyperplasia, mild short spindle cells with obvious vascularized stroma, and immunohistochemical testing results that are usually not characteristic.^[10]^ The histological features of prostatic stromal sarcoma are characterized by spindle or epithelioid tumor cells arranged in solid or fascicular patterns, accompanied by significant cellular atypia, mitotic figures, and tumor necrosis. Immunohistochemical staining results show that tumor cells usually express vimentin, CD34, and PR, and to varying degrees express SMA and desmin.^[11]^ In terms of histomorphology of prostatic synovial sarcoma, the tumor cells can present as spindle-shaped, arranged in fascicles, or epithelioid, forming tubular or trabecular structures, with frequent mitotic figures. According to the proportion of 2 forms, synovial sarcoma can be classified into monophasic, biphasic, and poorly differentiated types. Immunohistochemically, tumor cells express TLE1, EMA, CK19, and some cases could express S-100, CD99, and Bcl-2. Molecular genetic studies confirmed that tumors had a *t*(*X*; 18; p11; q11) translocation or SYT-SSX gene fusion transcript.^[12,13]^ The immunohistochemical examination results of our 3 patients are relatively consistent with the findings of the aforementioned study. For the first patient, we initially considered the possibility of STUMP. However, after conducting a detailed analysis of the patient’s histomorphology and immunohistochemistry, we found that the patient lacked the pathological and immunohistochemical features associated with STUMP as defined in the 2022 WHO classification. Therefore, we are more inclined to diagnose the patient with prostatic interstitial smooth muscle nodular hyperplasia. For the second patient, the pathological and immunohistochemical diagnostic results revealed characteristics consistent with a mesenchymal malignant tumor. To differentiate it from synovial sarcoma, we performed SS18 gene testing on the patient, as the detection of SS18 gene translocation is considered the gold standard for diagnosing synovial sarcoma. However, the test results for this patient were negative. Although this result does not completely rule out the diagnosis of synovial sarcoma, it is insufficient to support it. Consequently, when SS18 gene translocation testing yields negative results, our diagnosis relies more heavily on the patient’s morphological characteristics and immunohistochemical findings. After comprehensive consideration, we ultimately concluded that the patient had prostate stromal sarcoma. In the case of the third patient, the SS18 gene test demonstrated a clear translocation, leading to a diagnosis of prostate synovial sarcoma.

Treatment options for spindle cell lesions of the prostate depend on the type of lesion and the specific situation of the patient. For benign lesions such as prostatic stromal nodules of hyperplasia, if the patient has obvious clinical symptoms, we can perform transurethral laser-induced prostatectomy. The prognosis for the patient is generally satisfactory, and our patient has shown no obvious signs of recurrence so far. For malignant lesions, whether it is prostatic stromal sarcoma or prostatic synovial sarcoma, although there is currently no standardized treatment strategy, complete surgical resection is still the most effective treatment method when conditions permit.^[14]^ The main surgical methods are radical prostatectomy, radical cystoprostatectomy and total pelvic exenteration.^[15]^ For patients with tumors larger than 5 cm in diameter, tumors adjacent to critical neurovascular structures, and distant metastases, it is recommended that radiotherapy or chemotherapy be performed first to reduce the tumor before resection.^[16]^ What needs to be pointed out here is that although the patient has already developed distant metastases, surgical treatment should still be preferentially considered. Generally, other treatment methods are required after surgery in order to maximize the benefits to the patient. The main options include radiotherapy, chemotherapy, targeted therapy, immunotherapy, etc.^[17]^ Radiotherapy is generally not used alone and needs to be combined with surgery or chemotherapy. Currently, radiotherapy is available using 90Y-FAPI-46.^[18]^ Commonly used chemotherapeutic drugs include actinomycin D, vincristine, cyclophosphamide and doxorubicin. The current primary chemotherapy regimen is to use anthracyclines alone or in combination with ifosfamide.^[19]^ Other Studies have shown that using adriamycin in combination with ifosfamide as adjuvant chemotherapy has prevented tumor recurrence for over 10 months.^[20]^ Sakura et al used a chemotherapy regimen consisting of etoposide, ifosfamide and cisplatin, which has been shown to be effective for patients who developed lymph node metastasis 5 months after radical prostatectomy, and no recurrence was observed during a 4-year follow-up period.^[21]^ Molecularly targeted drugs, tyrosine kinase inhibitors Pazopanib and Anlotinib may significantly improve the prognosis of patients with prostatic sarcoma.^[22,23]^ Researchers have revealed that defects in homologous recombination repair may be another innovative target for the treatment of prostatic sarcoma.^[24]^ NY-ESO-1 immunotherapy has also emerged as a possible new treatment for sarcoma. Large-scale clinical trials are still needed to evaluate whether it can improve patient outcomes.^[25]^ For some patients with advanced tumors or those who are not physically able to undergo surgery, multiple modalities described above can be chosen for combination therapy. Current research also shows that transcatheter arterial chemoembolization for advanced prostatic sarcoma also has a good therapeutic effect and can significantly alleviate the clinical symptoms of patients.^[26]^ The second patient refused chemotherapy for personal reasons and chose anlotinib as a targeted therapy. Unfortunately, after 3 months of treatment, the patient passed away. In contrast, the third patient received a combination of Liposome doxorubicin and ifosfamide, which had a significant effect. The patient’s tumor volume shrank significantly, and the symptoms of dysuresia were also greatly relieved. It is currently planned that after 4 cycles of chemotherapy, the patient’s eligibility for surgery will be reevaluated.

Spindle cell lesions of the prostate are a complex group of lesions, and their diagnosis requires a comprehensive judgment based on imaging tests, pathological features, immunohistochemical staining, and even genetic test results. For malignant lesions, such as prostatic sarcoma, the prognosis is usually poor due to their high metastatic potential and recurrence rate. Once diagnosed, comprehensive treatment with an emphasis on surgery needs to be performed as soon as possible if conditions permit. While this study serves as a reference for the clinical diagnosis and treatment of prostate spindle cell lesions, it is important to acknowledge several limitations. Firstly, the study involved only 3 patients, which resulted in a small sample size and considerable heterogeneity among cases. This limitation may hinder the results from accurately representing the overall characteristics of this disease. Secondly, the follow-up period was relatively short, particularly for cases involving malignant tumors, and there was a lack of long-term survival data. Consequently, it is challenging to draw definitive conclusions regarding the long-term efficacy of treatment regimens or patient outcomes. Furthermore, the treatment strategies varied across cases, and no control group was established, which further restricts the reliability of efficacy comparisons. Future research should aim for larger, multicenter studies to more comprehensively evaluate the biological behavior of prostate spindle cell lesions and the impact of various treatment options.

## Author contributions

**Conception and design:** Quanxi Wang, Ke Dou.

**Administrative support:** Ke Dou.

**Provision of study materials or patients:** Quanxi Wang, Peng Li.

**Collection and assembly of data:** Zhengdong Zong, Peng Li.

**Data analysis and interpretation:** Quanxi Wang, Zhengdong Zong.

**Writing** – **original draft:** Quanxi Wang, Peng Li, Zhengdong Zong, Ke Dou.

**Writing** – **review & editing:** Ke Dou.
